# Synergistic effect of collagenase-1 (MMP1), stromelysin-1 (MMP3) and gelatinase-B (MMP9) gene polymorphisms in breast cancer

**DOI:** 10.1371/journal.pone.0184448

**Published:** 2017-09-29

**Authors:** Chiranjeevi Padala, Mohini Aiyengar Tupurani, Kaushik Puranam, Srilatha Gantala, Nivas Shyamala, Mrudula Spurthi Kondapalli, Kishore kumar Gundapaneni, Saraswati Mudigonda, Rajesh Kumar Galimudi, Keerthi Kupsal, Santoshi Rani Nanchari, Uday Chavan, Sanjeeva kumari Chinta, Srinivasulu Mukta, Vishnupriya Satti, Surekha Rani Hanumanth

**Affiliations:** 1 Department of Genetics, Osmania University, Hyderabad, Telangana State, India; 2 MNJ Institute of Oncology & Regional Cancer Centre, Red Hills, Hyderabad, Telangana State, India; University of South Alabama Mitchell Cancer Institute, UNITED STATES

## Abstract

**Background:**

Extracellular matrix degradation by matrix metalloproteinases (MMPs) is an important mechanism involved in tumor invasion and metastasis. Genetic variations of MMPs have shown association with multiple cancers. The present study is focused to elucidate the association of MMP-1, 3 and 9 genetic variants with respect to epidemiological and clinicopathological variables by haplotype, LD, MDR, survival in silico analyses among South Indian women.

**Material and methods:**

MMP3–1171 5A/6A and MMP9–1562 C/T SNPs were genotyped by Allele specific polymerase chain reaction and MMP1-1607 1G/2G polymorphism by restriction fragment length polymorphism assays respectively, in 300 BC patients and age-matched 300 healthy controls. Statistical analysis was performed using the SNPStats and SPSS software. Linkage disequilibrium and gene-gene interactions were performed using Haploview and MDR software respectively. Further, transcription factor binding sites in the promoter regions of SNPs under study were carried out using AliBaba2.1 software.

**Results:**

We have observed an increased frequency of 2G-allele of MMP1, 6A-allele of MMP3 and T-allele of MMP9 (p<0.05) respectively in BC subjects. The 2G-6A haplotype (minor alleles of MMP-1 and MMP-3 respectively) has shown an increased susceptibility to BC. Further, MMP polymorphisms were associated with the clinical characteristics of BC patients such as steroid hormone receptor status, lymph node involvement and metastasis. SNP combinations were in perfect LD in controls. MDR analysis revealed a positive interaction between the SNPs. 5-years survival rate and cox-regression analysis showed a significant association with clinicopathological variables.

**Conclusion:**

Our results suggest that MMP1–1607 1G/2G, MMP3–1171 5A/6A and MMP9–1562 C/T gene polymorphisms have synergistic effect on breast cancer. The interactions of MMPs clinical risk factors such as lymph node involvement has shown a strong correlation and might influence the 5-years survival rate, suggesting their potential role in the breast carcinogenesis.

## Introduction

Breast Cancer (BC) is the most common cancer and a leading cause of death in women worldwide and in India. It is a multi-factorial, polygenic disease resulting from the interplay of genetic, epigenetic, environmental and lifestyle factors [1]. The breast microenvironment composed of extracellular matrix (ECM), stromal cells including endothelial and immune cells, fibroblasts and adipocytes playing a crucial role in mammary duct morphogenesis. Key enzymes regulating ECM turnover are matrix metalloproteinases (MMPs) and tissue inhibitors of metalloproteinases (TIMPs) [2].

The matrix metalloproteinases are a family of secreted Zn-dependent endopeptidases playing an important role in the physiological processes and its deregulation is associated with various diseases including cancers [3]. The deregulated MMPs results in cancer progression such as cell proliferation, angiogenesis, invasion [4], metastasis and escape from the immune surveillance [5–6]. Activation of MMPs can be controlled by proteolytic enzymes such as plasmin, while their inhibition is controlled by their specific endogenous TIMPs [7].

MMPs are broadly divided into collagenases (MMP1), stromelysins (MMP3), gelatinases (MMP9) and membrane-associated-MMPs on the basis of their substrate specificity. Collagenases include MMP1, one of the most widely expressed MMP and can degrade type I, II, and III collagens. Stromelysins include MMP-3 an enzyme capable of degrading laminin, fibronectin, gelatins of type I, III, IV, and V; collagens and cartilage proteoglycans [8]. Gelatinase-B or MMP9 digest gelatins or denatured collagens [9].

The progress in the knowledge about the role of MMPs and their inhibitors in tumourigenesis have led to numerous studies which have tested a potential association of single nucleotide polymorphisms (SNPs) in these genes with cancer susceptibility and progression [10,11]. Although research has been performed to explore the role of SNPs of these genes in breast cancer individually [12–16], the results are inconclusive. Therefore, the present study has been performed to derive a more precise estimation of the association of MMPs and to elucidate the synergistic effect of genetic polymorphisms in the regulatory regions of MMP1 (rs1799750), MMP3 (rs35068180) and MMP9 (rs3918242) for susceptibility and progression of breast cancer by analysis of SNPs, haplotypes, LD, MDR, survival and in silico analysis along with epidemiological and clinicopathological variables in South Indian women.

## Materials and methods

### Ethics statement

This case-control study was carried out with the approval of the Ethics Committee of Mehdi Nawaj Jung (MNJ) Institute of Oncology & Regional Cancer Centre, Hyderabad, Telangana State, INDIA. The subject recruitment and sample collection were done only after obtaining written informed consent from the participants.

### Study subjects

The present study consists of 300 confirmed breast cancer patients and 300 healthy controls from South India. Patients with breast cancer were consecutively recruited from MNJ regional cancer center, Hyderabad and women with any other cancer or other systemic inflammatory disease were excluded from the case and control group. Patients were enrolled from the department of oncology between the period from August 2011 to August 2016. Selection criteria for cases included patients who were histopathologically confirmed as breast cancer by medical and surgical oncologists. The patients were subjected to detailed demographical, clinical and pathological investigations. Staging of cancer was documented according to the AJCC-TNM classification system.

During the same time the control group was drawn from the same region with similar socio-economic status and the individuals included had no evidence of any personal history of cancer or other malignant conditions. General health history of the controls was collected with an appropriately designed proforma.

### Data collection

A detailed description of the baseline characteristics of the breast cancer patients and healthy controls is shown in Table 1.

**Table 1:** Baseline characteristics in controls and breast cancer patients.

Characteristics	Controls N = 300/n (%)	Cases N = 300/n (%)
**Demographic factors**		
**Age (years)**	46.34±7.97	47.79±10.35
**≤40**	82 (27.33)	87(29.0)
**>40**	218(72.66)	213(71.0)
**Age at menarche(years)**	14.01±1.61	13.07±1.13
**Age at menopause (years)**^a^	47.7±4.8	48.4±4.5
**Area of Living**		
**Rural**	171(57.0)	188(62.66)
**Urban**	129(43.0)	112(37.33)
**Menstrual cycles**		
**Regular**	247(82.33)	236(78.66)
**Irregular**	53(17.66)	64(21.33)
**Mensuration status**		
**Premenopause**	156(52.0)	176(58.66)
**Postmenopause**	144(48.0)	124(41.33)
**Lifestyle risk factors**		
**Smoking Habit**		
**Smoker**	27(9.0)	55(18.66)
**Non-smoker**	273(91.0)	245(81.7)

Continuous variables: Mean±SD, Categorical variables: Numbers,

^a^ Among Postmenopause women.

Clinical profile of breast cancer was evaluated with the help of medical and surgical oncologists according to the Union for International Cancer Control (UICC) and tumor–node metastasis (TNM) classification for breast cancer (WHO) and the same was noted in the case proforma from the tumor registries as shown in Table 2. [17].

**Table 2:** Clinicopathological parameters among the breast cancer patients.

Parameters	Breast cancer patients	
	Number	Percentage
**Stage of the cancer**		
**T**_**0**_**-T**_**2**_	197	65.7
**T**_**3**_**-T**_**4**_	103	34.3
**ER**^a^ **status**		
**Positive**	177	59.0
**Negative**	123	41.0
**PgR**^b^ **status**		
**Positive**	168	56.0
**Negative**	132	44.0
**HER2/neu**^c^ **receptor status**		
**Positive**	163	54.33
**Negative**	111	37.0
**Missing Data**	26	8.6
**Triple Negative Receptor status**		
**Other combinations**	215	71.66
**Negative**	59	19.67
**Missing Data**	26	8.67
**Histological subtype**		
**Ductal Carcinoma**	247	82.33
**Lobular Carcinoma**	53	17.66
**Lymph nodal status**		
**Positive**	220	73.33
**Negative**	80	26.66
**Metastasis**		
**Absent**	198	66.0
**Present**	102	34.0

^a^Oestrogen receptor

^b^Progesterone receptor

^c^Human epidermal growth factor receptor 2 /neu receptor

### Genomic DNA extraction

From each subject 4ml of blood was drawn into vaccutainer tubes containing ethylene-di-amine-tetra-acetic acid (EDTA) and stored at 4°C. Genomic DNA was extracted from the whole blood sample by using non-enzymatic salting out method [18].

### Genotyping

The promoter SNPs (Single nucleotide polymorphisms) in the MMP1, MMP3 and MMP9 genes were genotyped using PCR-RFLP (Restriction Fragment Length Polymorphism) method and AS-PCR (Allele Specific-Polymerase Chain Reaction) assays [16,19,20].

For confirmation, genotyping was performed without the knowledge of subjects case/control status. Furthermore, in order to ensure the accuracy of the genotyping data, our data was confirmed by Sanger sequencing analysis and the results were found to be in 100% concordance.

### Statistical analysis

The continuous variables are expressed as the mean ± standard deviation (Mean±SD). Chi-square test for goodness-of-fit was used to analyze the difference in the frequency distribution between cases and controls for discontinuous variables. Hardy-Weinberg equilibrium test was performed between controls and patients for each SNP. The allele and genotype frequencies for all the polymorphisms were calculated using Chi-Square [χ^2^] test for significance of differences between cases and controls. Adjusted odds (AOR) ratios were calculated by adjusting covariates such as age and haplotype frequencies were estimated in controls and cases using SNPStats [21]. Linkage disequilibrium (LD) plots of controls and cases were generated using Haploview program [22]. Gene–gene interactions were determined by MDR analysis [23]. We have also compared the allele and genotype distribution for all clinical and histopathological characteristics of cases. The p-values <0.05 were considered statistically significant.

The Kaplan-Meier survival analysis was carried out on the follow up data available from 216 breast cancer cases, taking death as an event occurring within 5 years of diagnosis to calculate median 5-years survival (OS) rate. The multivariate analysis of the probable predictive factors for survival was carried out using Cox's proportional hazard regression analysis.

### In silico analysis

The SNPs in the promoter regions of MMP-1, -3 and -9 genes were studied for the presence of transcription factor binding sites using AliBaba2.1 online tool (http://www.gene-regulation.com/pub/programs/alibaba2/index.html).

## Results

### Characteristics of study population

The baseline characteristics of controls and patients are listed in Table 1. The age of the controls ranged from 35 to 85 years with a mean age of 46.34±7.97, and the age of the breast cancer patients was within a range of 36–86 years, and their average age at diagnosis was 47.79 ±10.35 years respectively. According to the baseline characteristics, there was no significant difference in terms of epidemiological variables between cases and controls. However, a higher number of breast cancer patients (62.6% versus 57.0%) were from rural background compared to controls. More than 75% had regular menstrual cycles and about 176 (58.6%) patients were in premenopausal state and 124 (41.33%) in postmenopausal state. About 18.3% of breast cancer patients had smoking habit as presented in Table 1.

In the present study, clinicopathological profiles of breast cancer patients revealed that majority of the patients (66%) were at clinical stage T_0_-T_2_ (early stage)and with respect to information on steroid hormone receptor status, out of total patients, 176 (58.6%) patients were ER positive, 167 (55.6%) were PgR positive and 163 (54.33%) Her2/nu positive whereas 59 (19.66%) were triple negative status. The histopathological classification of breast cancer at the time of diagnosis in our study showed that of all patients, 82.33% (247) had ductal carcinoma and 17.66% (53), had invasive lobular carcinoma. 73.33% of the patients had shown positive lymph node involvement at the time of diagnosis. Among the total cases, 102 (34%) reported metastasis as presented in Table 2. The SNPs of MMP1 (-1607 1G/2G), MMP3 (-1171 5A/6A) and MMP9 (-1562 C/T) were genotyped using specific primers and thermal cycling conditions as displayed in Table 3.

**Table 3:** Forward and reverse primers and PCR conditions for genotyping of selected polymorphic variants of MMP-1,-3 & -9 genes.

SNP rsnumber	Assay	Primers	Ta	RE	Gel band pattern	Ref.
-1607 1G/2G (rs1799750)	PCR-RFLP	5'-gacttttaaaacatagtctatgttca-3' (f)	52°C	*Alu I*	1G- 241bp, 28bp	[16]
		5'-tcttggattgatttgagataagtcatagc-3' (r)			2G-269-bp	
-1171 5A/6A (rs35068180)	AS-PCR	5A-ttgatggggggaaaaac (f)	54°C	-	5A- 226bp	[19]
		5A-actccagagaaaatttacaaagg (r)				
		6A-ttgatggggggaaaaaa (f)			6A- 282bp	
		6A-aacatattatctatcaggctttcct(r)				
-1562 C/T (rs3918242)	AS-PCR	CF-5′aaaaatttagccaggcgtggtggcgaac 3'	58°C	-	C-296bp	[20]
		CR- 5′ctcccgagtagctggtattatagtca 3'				
		TF 5′aaatttagccaggcgtggtggcga at			T-220bp	
		TR 5′gcctcccgagtagctggtattatagtc g 3'				

f-forward; r-reverse; Ta–Annealing temperature, RE- restriction Enzyme, AS-Allele Specific Ref-reference

The distribution of genotype and allele frequencies of SNPs under study for 300 controls and 300 breast cancer patients is shown in Table 4 (S1 File).

**Table 4:** Genotype and allele frequencies distribution for MMP-1, MMP-3 and MMP-9 gene polymorphisms in controls and breast cancer subjects.

MMP1–1607 1G>2G	Controls	BC Cases	OR (95%CI)	p-value
**1G1G**	66 (22)	26 (8.6)	1.0(ref)	
**1G2G**	122 (40.7)	108 (36)	2.21(0.92–3.77)	
**2G2G**	112 (37.3)	166(55.3)	4.13(2.14–7.00)	<0.01*
**1G**	254(0.42)	160(0.27)		
**2G**	346(0.58)	440(0.73)	2.01(1.57–2.59)	<0.0001*
**MMP3–1171 5A>6A**				
**5A5A**	88 (29.3)	59 (19.7)		
**5A6A**	171 (57)	189 (63)	1.65 (1.12–2.43)	0.011*
**6A6A**	41 (13.7)	52 (17.3)	1.89 (1.12–3.2)	0.023*
**5A**	347 (0.58)	347 (0.58)		
**6A**	253 (0.42)	253 (0.42)	1.3 (1.04–1.64)	0.02*
**MMP9–1562 C>T**				
**CC**	150 (50)	121 (40.3)		
**CT**	101 (33.7)	107 (35.7)	1.31 (0.91–1.88)	0.07
**TT**	49 (16.3)	72 (24)	1.82 (1.18–2.81)	0.02*
**C**	401 (0.67)	349 (0.58)		
**T**	199 (0.33)	251 (0.42)	1.44 (1.14–1.83)	0.0001*

*p<0.05 is considered to be statistically significant

### Distribution of allelic and genotype frequencies of 1G -1607 2G polymorphism in MMP1 gene

The distribution of allelic frequencies of MMP1–1607 1G and 2G alleles were found to be 0.42 and 0.58 in controls, compared with 0.27 and 0.73 in patients respectively, thus an increased frequency of 2G-allele of MMP1 (OR 2.01, 95%CI 1.57–2.59, p<0.001) was observed in BC subjects (*p*<0.01) as summarized in Table 4. The genotype distribution in controls were 22% (1G/1G), 40.7% (1G/2G), and 37.3% (2G/2G), while in patients were 8.7% (1G/1G), 36.0% (1G/2G) and 55.3% (2G/2G), respectively. The frequency distribution in patients had shown slight deviation from those expected from the Hardy Weinberg equilibrium (*p* = 0.18 and 0.04 for controls and cases respectively). The frequency of variant homozygote (2G/2G) was found to be significantly high in the BC group compared to controls with 4 folds increased risk for BC (OR 95% CI 4.13, 2.14–7.00, *p*<0.0001).

### Distribution of allelic and genotype frequencies of 5A -1171 6A polymorphism in MMP3 gene

The distribution of allelic frequencies of MMP3–1171 5A and 6A alleles were found to be 0.58 and 0.42 in controls, compared with 0.51 and 0.49 in patients respectively, showing an increased frequency of 6A-allele of MMP3 (OR 1.30, 95% CI 1.04–1.64, p<0.023 in BC patients (*p* = 0.023) as summarized in Table 4. The frequencies of the three MMP3 genotypes in controls were 29.3% (5A/5A), 57.0% (5A/6A) and 13.7% (6A/6A) while, in BC patients the frequencies were 19.7% (5A/5A), 63% (5A/6A) and 17.3% (6A/6A) and there was a deviation from those expected from the Hardy Weinberg equilibrium (*p* = 0.004, *p* = 0.0001, for controls and cases respectively). The frequency of homozygote variant (6A/6A) and heterozygote 5A/6A were found to be significantly high in the BC group compared to controls with 1.89 and 1.65 fold increased risk for BC (OR 95% CI 1.89 1.12–3.20, *p* = 0.01, OR 95% CI 1.65, 1.12–2.43 p = 0.01).

### Distribution of allelic and genotype frequencies of C-1562 T polymorphisms in MMP9 gene

The distribution of allelic frequencies of MMP9–1562 C and T alleles were found to be 0.67 and 0.33 in controls, compared with 0.58 and 0.42 in patients respectively, with an increased frequency of T-allele of MMP9 (OR 1.44, 95% CI 1.14–1.83, p<0.0023) was observed in BC patients as summarized in Table 4. The frequencies of the three MMP9 genotypes in controls were 50% (C/C), 33.7% (C/T) and 16.3% (T/T) while, in BC patients were 40.3% (C/C), 35.7% (C/T) and 24% (T/T), deviated from those expected from the Hardy Weinberg equilibrium (*p*<0.01). The frequency of variant homozygote (T/T) was found to be higher in the BC group compared to controls with 1.82 fold increased risk for BC (OR 95% CI 1.82 1.18–2.81, *p* = 0.02).

### Stratified analysis of MMP1–1607 1G/2G, MMP3–1171 5A/6A and MMP9–1562 C/T gene polymorphisms in association with clinicopathological features

Genotype distribution of MMP1–1607 1G/2G polymorphism with respect to clinical characteristics is summarized in Table 5. It has been observed that the frequency of individuals with 1G allele was high with HER2/neu receptor negative status indicating protection against the disease. The other variables like stage of cancer, histological type of tumor, ER/PR status, lymph node status and metastasis status have not revealed any significant association with MMP-1–1607 1G/2G polymorphism.

**Table 5:** Association of the MMP1–1607 1G>2G, MMP3-1171 5A>6A and MMP9-1562 C>T genotypes with breast cancer susceptibility and clinicopathological characteristics.

Characteristics	MMP1–1607 1G>2G			MMP3-1171 5A>6A			MMP9-1562 C>T		
	1G1G	1G2G	2G2G	5A5A	5A6A	6A6A	CC	CT	TT
Stage of the cancer									
Early	17 (8.6)	75 (38.1)	105 (53.3)	66 (33.5)	83(42.1)	48(24.4)	79(40.1)	69 (35.0)	49 (24.9)
Late	9 (8.7)	33 (32)	61 (59.2)	43(41.8)	37 (35.9)	23(22.3)	42(40.8)	38(36.9)	23 (22.3)
OR (95% CI)	1.0(ref)	0.84 (0.34–2.08)	1.09 (0.46–2.6)	1.00	0.68(0.40–1.18)	0.74(0.39–1.39)	1.0(ref)	1.03(0.59–1.77)	0.88(0.47–1.64)
p-value		0.61	0.83	1.0(ref)	0.37	0.33		0.89	0.69
Type of the cancer									
Ductal	21 (8.5)	96 (38.9)	130 (52.6)	47(19.0)	158(64.0)	42(17.0)	96(38.9)	89(36.0)	62(25.1)
Lobular	5 (9.4)	12 (22.6)	36 (67.9)	12(22.6)	31(58.5)	10(18.9)	25(47.2)	18(34.0)	10(18.9)
OR (95% CI)	1.0(ref)	0.56 (0.18–1.77)	1.12(0.39–3.21)	1.0(ref)	0.76(0.36–1.60)	0.93(0.36–2.37)	1.0(ref)	0.76(0.98–1.48)	0.62(0.28–1.37)
p-value		0.14	0.77		0.74	0.88		0.45	0.23
Axillary Lymph node Status									
Positive	19 (8.6)	77 (35)	124 (56.4)	34(15.4)	147(66.8)	39(17.7)	88(40.0)	79(35.9)	53(24.1)
Negative	7 (8.8)	31 (38.8)	42 (52.5)	25(31.2)	42(52.5)	13(6.2)	33(41.2)	28(35.0)	19(23.8)
OR (95% CI)	1.0(ref)	0.91(0.35–2.39)	1.09(0.43–2.78)	1.0(ref)	**2.58(1.39–4.80)**	2.20(0.97–4.96)	1.0(ref)	1.07(0.59–1.93)	1.04(0.54–2.02)
p-value		0.82	0.86		0.01*	0.05		0.85	0.89
ER Status									
Positive	16(9.0)	66(37.3)	95(53.7)	35(19.8)	110(62.1)	32(18.1)	73(41.2)	62(35.0)	42(23.7)
Negative	10(8.1)	42(34.1)	71(57.7)	24(19.5)	79(64.2)	20(16.3)	48(39.0)	45(36.6)	30(24.4)
OR (95% CI)	1.0(ref)	1.01(0.42–2.45)	1.20(0.59–3.80)	1.0(ref)	1.05(0.58–1.90)	0.91(0.42–1.95)	1.0(ref)	1.11(0.65–1.89)	1.09(0.60–1.97)
p-value		0.77	0.67		0.91	0.8		0.92	0.78
PgR Status									
Positive	16 (9.6)	62 (37.1)	89 (53.3)	34(20.2)	102(60.7)	32(19.1)	16(9.6)	62(37.1)	89(53.3)
Negative	10 (7.5)	46 (34.6)	77 (57.9)	25(18.9)	87(65.9)	20(19.1)	10(7.5)	46(34.6)	77(57.9)
OR (95% CI)	1.0(ref)	1.18 (0.49–2.85)	1.39(0.59–3.24)	1.0(ref)	1.16(0.64–2.09)	0.85(0.40–1.82)	1.0(ref)	1.18(0.49–2.85)	1.39(0.59–3.24)
p-value		0.67	0.45		0.6	0.67		0.67	0.45
HER2/neu Status									
Positive	11 (6.8)	45 (24.6)	107 (65.6)	36(22.1)	98(60.1)	29(17.8)	63(38.6)	54(33.1)	46(28.2)
Negative	12 (10.8)	49 (44.1)	50 (45)	17(15.3)	73(65.8)	21(18.9)	47(42.3)	42(37.8)	22(19.8)
OR (95% CI)	1.0(ref)	1.0(0.40–2.49)	0.43(0.18–1.04)	1.0(ref)	1.58(0.82–3.03)	1.53(0.69–3.34)	1.0(ref)	1.04(0.60–1.81)	0.64(0.34–1.21)
p-value		0.003	0.05		0.34	0.29		0.88	0.16
Distant Metastasis Status									
Positive	10(9.8)	36(35.3)	56(54.9)	19(18.6)	64(62.8)	19(18.6)	37(36.3)	38(37.2)	27(26.5)
Negative	16 (8.1)	72(36.4)	110(55.6)	40(20.2)	125(63.1)	33(16.7)	84(42.4)	69(34.9)	45(22.7)
OR (95% CI)	1.0(ref)	0.79(0.33–1.92)	0.82(0.35–1.93)	1.0(ref)	1.08(0.58–2.02)	1.21(0.55–2.65)	1.0(ref)	1.27(0.73–2.51)	1.36(0.72–2.51)
p-value		0.87	0.63		0.86	0.63		0.56	0.32

*p<0.05 is considered to be statistically significant

However, 5A/6A genotype of MMP3–1171 5A/6A polymorphism was found to be significantly associated with lymph node positive cases (OR 2.58, 95%CI 1.39–4.8, *p* = 0.01). There was no significant association of MMP9–1562 C/T gene polymorphism with respect to clinicopathological variables as summarized in Table 5 (S1 File).

### Haplotype analysis

Haplotype analysis of MMP1 and MMP3 genes present on chromosome 11q was performed to calculate the combined effect of MMP1–1607 1G/2G and MMP3–1171 5A/6A polymorphisms on breast cancer. Out of the four haplotypes obtained, the 2G (MMP1)-5A (MMP3) was the most commonly found haplotype therefore was considered as reference. The haplotype 1G-5A (alleles in order of MMP-1 & MMP-3) frequency was high in controls than in patients and might confer protection against breast cancer (OR 0.45, 95% CI 0.29–0.69, p<0.0001) Table 6 (S1 File).

**Table 6:** Distribution frequency of MMP-1 and MMP-3 haplotypes in healthy control subjects and BC patients.

Variable		Controls	Cases	Total Frequency	OR (95% CI)	*p*-Value
MMP1-1607 1G/2G	MMP3-1171 5A/6A					
**2G**	**5A**	0.3535	0.3836	0.3535	1.00	19
**2G**	**6A**	0.3015	0.3497	0.655	1.28(0.89–1.83)	
**1G**	**5A**	0.1915	0.1281	0.8465	**0.45(0.29–0.69)**	<0.0001*
**1G**	**6A**	0.1535	0.1386	1.0	0.74(0.49–1.11)	0.14
**Global haplotype association p-value: <0.0001**						

*p<0.05 is considered to be statistically significant

### Linkage disequilibrium

**Fig 1:**
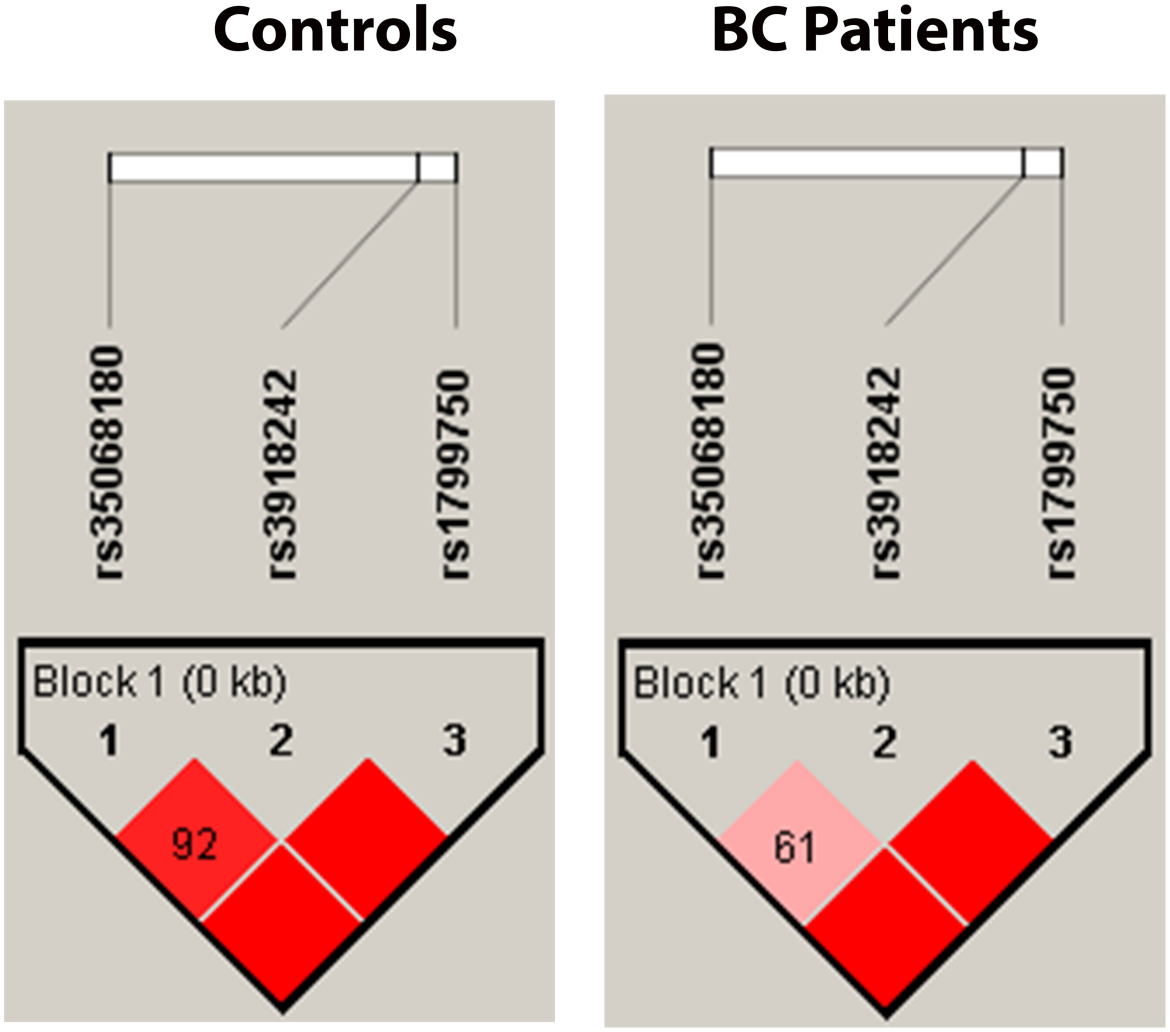
Linkage disequilibrium plot of three SNPs of the MMP1, MMP3 and MMP9 gene in controls and breast cancer patients.

In the present study, pairwise LD estimate was obtained for the MMP1, MMP3 and MMP9 gene polymorphisms in cases and control group separately. The analysis revealed that most of the SNP marker combinations exhibited perfect LD scores, with the exception of few combinations that showed differential pattern of high LD scores in each of the analysis group (controls and cases). The SNP loci combination of MMP3-MMP1 and MMP9-MMP1 showed a perfect LD in controls and cases, however D′ = 0.92 and D′ = 0.61 was observed between MMP3- MMP9 in controls and cases respectively as shown in Table 7 and Fig 1 (S1 File).

**Table 7:** Pairwise linkage disequilibrium estimates in controls and cases group.

Controls	D'	^1^Lod score	r^2^
**MMP3:MMP9**	0.923	57.79	0.58
**MMP3:MMP1**	1.0	29.84	0.53
**MMP9:MMP1**	1.0	51.67	0.36
**Cases**			
**MMP3:MMP9**	0.618	21.23	0.288
**MMP3:MMP1**	1.0	36.24	0.34
**MMP9:MMP1**	1.0	26.71	0.26

^1^Lod score LOD was defined as log10 (L1/L0), where L1 = likelihood of the data under linkage disequilibrium, and L0 = likelihood of the data under linkage equilibrium. D′ was calculated as follows: D′ = D′ (normalized linkage disequilibrium measure) divided by the theoretical maximum for the observed allele frequencies.

### Multifactor dimensionality reduction (MDR) Analysis

**Fig 2:**
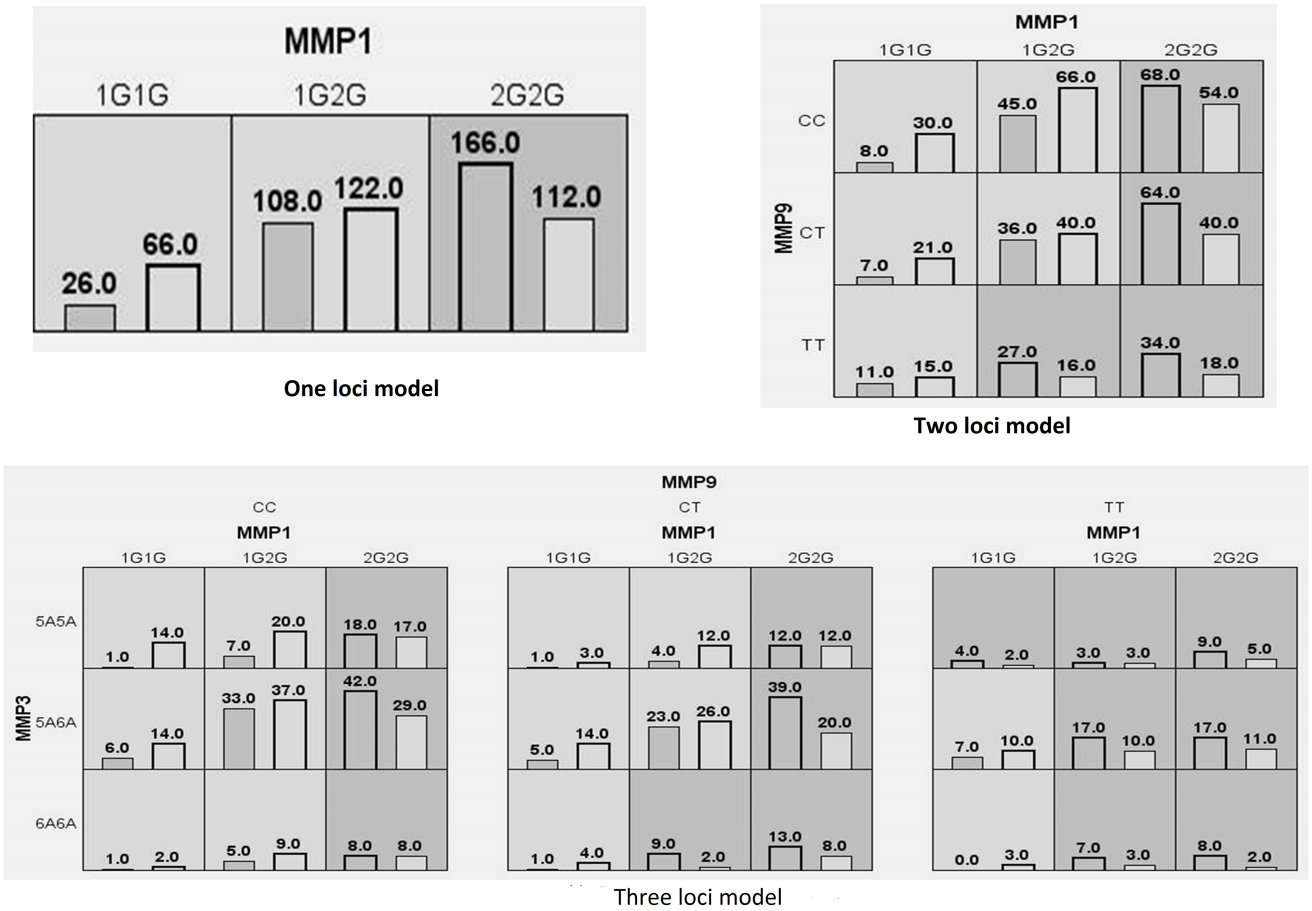
Summary of MDR gene-gene interaction.

Association of higher order interactions with breast cancer risk was analyzed by MDR analysis as summarized in Fig 2. The interaction information analysis revealed moderate effect between the markers -1607 1G/2G of MMP1, -1171 5A/6A of MMP3 and -1562 C/T of MMP9 genes which were conferring risk towards the progression of the breast cancer. High-risk and low-risk genotypic combinations were determined based on the threshold value, which was 1 (300/300) for the present data where the TT genotype of MMP9, 6A6A genotype of MMP3 and 2G2G genotype of MMP1 conferred a 3 fold high risk (8/2) for breast cancer as shown in Table 8 (S1 File).

**Table 8:** Summary of gene-gene interaction by MDR analysis.

Locus & Combinations	Genes	Training Balance Accuracy	Testing Error Balance Accuracy	p-value (sign test)	CVC Consistency
**One loci**	MMP1	0.59	0.41	8(0.054)	10/10
**Two loci**	MMP1,MMP9	0.61	0.39	8(0.054)	10/10
**Three loci**	MMP1, MMP3 and MMP9	0.61	0.39	7(0.171)	10/10

CVC = cross validation consistency

**Fig 3:**
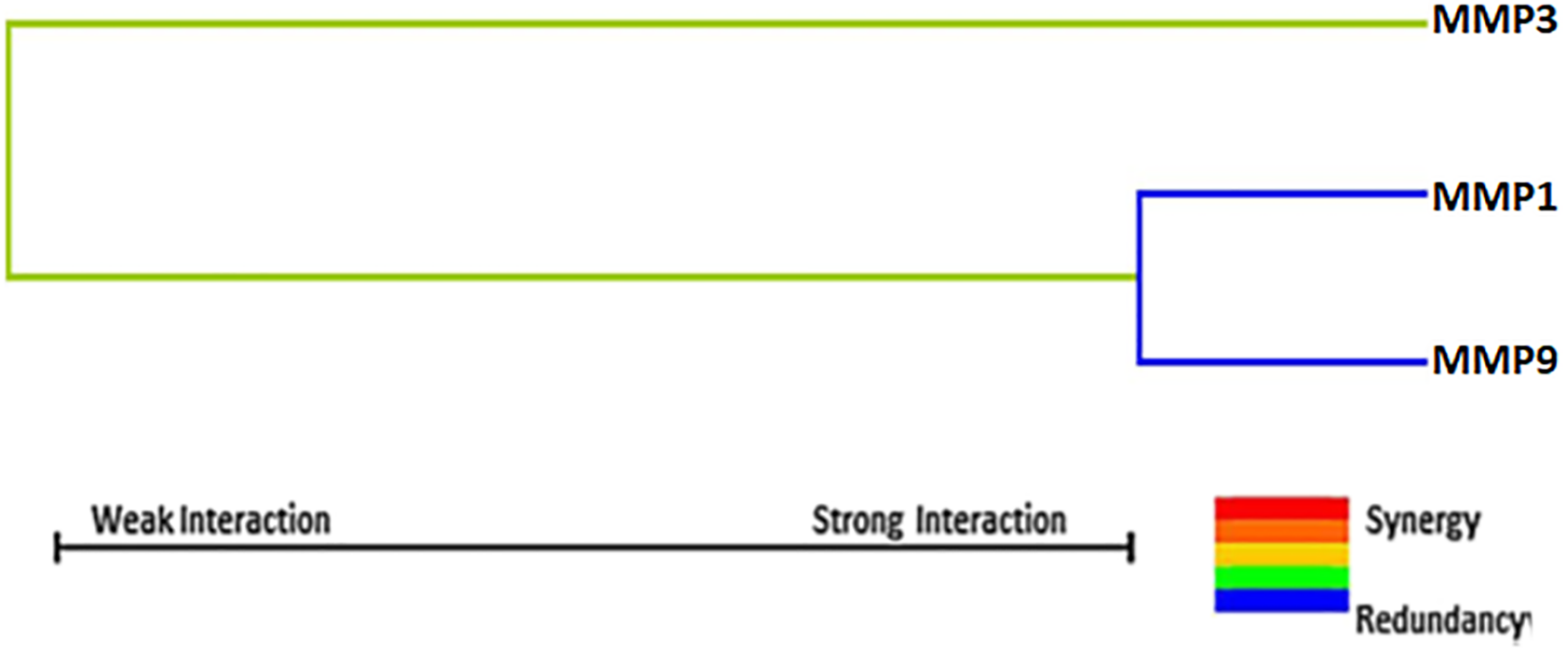
Interaction dendrogram of SNP-SNP by MDR analysis.

Dendrogram interaction analysis of our data showed that MMP1–1607 1G/2G and MMP9 -1562 C/T gene exhibited a strong redundant effect or correlation with the disease phenotype while MMP3–1171 5A/6A had an additive effect on the risk of breast cancer development as shown in Fig 3.

### Survival analysis

**Fig 4:**
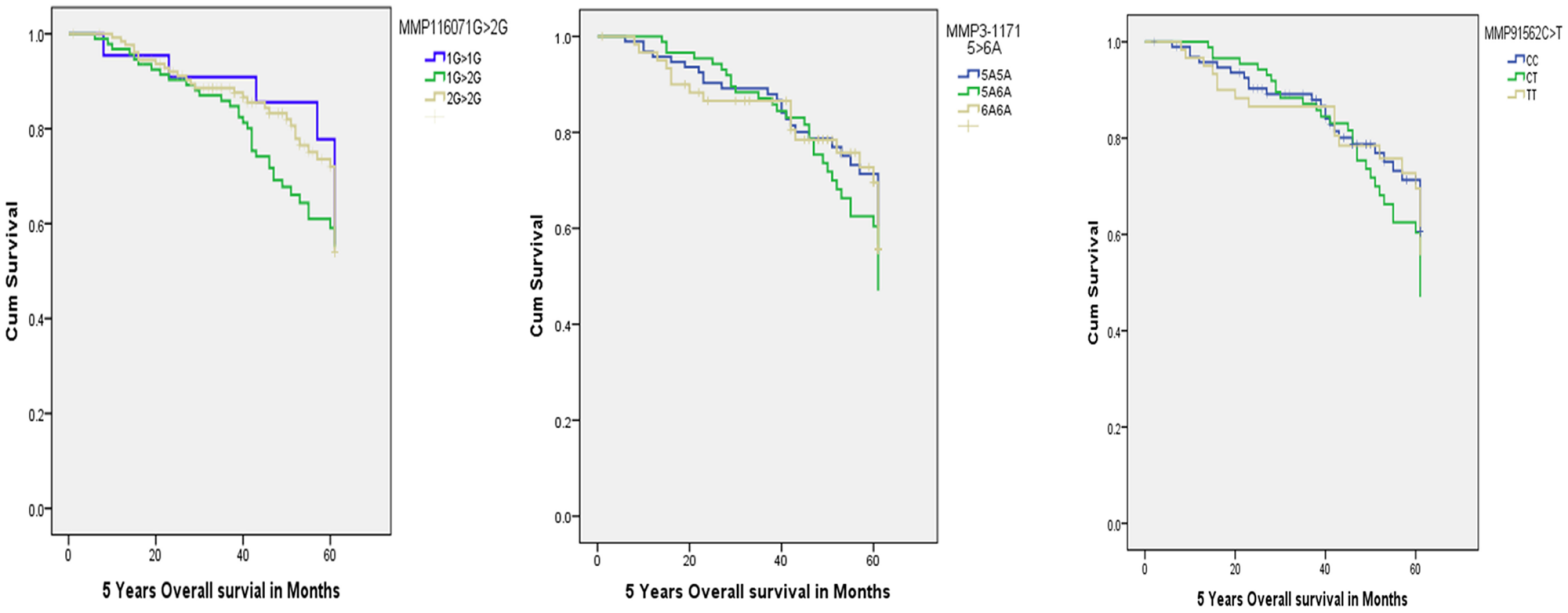
Kaplan-Meier 5 years survival analysis curves with respect to MMP1-16071G/2G, MMP3-1171 5A/6A and MMP9–1562 C/T polymorphisms in breast cancer patients.

The median survival rate was decreased in risk genotypes among all the selected SNPs of -1607 2G2G (48.0), -1171 6A6A (45.0) and -1562 TT (42.0) compared to protective genotypes of -1607 1G1G (50.0), -1171 5A5A (34.0) and -1562 CC (40.0) as summarized in Table 9. However, the genotypes did not significantly affect survival time among breast cancer patients (p>0.05) (Fig 4) (S2 File).

**Table 9:** Kaplan-Meier survival curve for 5- years survival rate in months of MMP1 (-1607 1G/2G), MMP3 (-1171 5A/6A) and MMP9 (-1562 C/T) polymorphisms.

Genotype	N (%)	Event (Death)	(5 years survival in months) Mean±SEM	Median	χ^2^ p-value^a^
**MMP1 (-1607 1G/2G) Gene Polymorphism**					
1G1G	22(8.94)	6(7.60)	55.590±3.35	50.00	0.51
1G2G	94(38.21)	35(44.30)	51.367±1.60	41.00	
2G2G	130(52.84)	38(48.10)	54.469±1.27	48.00	
Total	246	79	53.266±0.95	42.00	
**MMP3 (-1171 5A/6A) Gene Polymorphism**					
5A5A	46(18.67)	10(12.65)	54.924±2.22	34.00	0.09
5A6A	153(62.19)	49(62.02)	53.265±1.12	42.00	
6A6A	47(19.13)	20(25.33)	51.508±2.35	45.00	
Total	246	79	53.266±0.9	53.00	
**MMP9 (-1562 C/T) Gene Polymorphism**					
CC	96(39.02)	27(34.18)	53.630±1.56	40.00	0.495
CT	89(36.17)	33(41.80)	53.133±1.40	46.00	
TT	61(24.80)	19(24.01)	52.914±2.14	42.00	
Total	246	79	53.266±0.94	42.00	

^a^Log Rank p- values by mantle COX regression statistically significant at p<0.05

With respect to clinicopathological characteristics, our analysis revealed a significant association between median 5 year survival rate with late stage of breast cancer at diagnosis, negative HER2/neu receptor status and presence of metastasis (p<0.05).

In addition, the multivariate cox regression analysis also confirmed the results indicating the significant influence of the clinicopathological variables on breast cancer development and progression Table 10 (S2 File).

**Table 10:** Kaplan-Meier survival and cox regression analysis of clinicopathological characteristics on 5- years survival rate of breast cancer patients.

Characteristics	N (%)	Event (Death)	(5 years in months) Mean±SEM	Median	p-value	HR^a^	95% CI^b^	p-value
**Stage of the cancer**								
**Early Stage**	163 (66.26)	41(25.15)	134.30±8.07	59.0	0.002*	1(Ref)		0.003*
**Late stage**	83 (33.37)	38 (45.78)	79.61±7.32	48.0		1.962	1.26–3.05	
**Size of Tumor**								
**<50mm**	178(72.35)	52(29.21)	122.69±8.31	57.0	0.05*	1(Ref)		0.06
**>50mm**	68(27.64)	27(39.70)	74.47±5.54	47.0		0.63	0.39–1.07	
**Type of Cancer**								
**Ductal**	205 (83.34)	67 (32.69)	113.56±8.81	59.0	0.28	1(Ref)		0.26
**Lobular**	41 (16.66)	12 (29.27)	116.05±13.14	40.0		0.715	0.38–1.32	
**Lymph node status**								
**Lymph node -Ve**	61 (24.79)	17 (27.89)	133.70±11.80	47.0	0.69	1(Ref)		0.69
**Lymph node +Ve**	185 (75.21)	62 (33.51)	105.40±7.54	57.0		1.113	0.65–1.90	
**ER Status**								
**ER positive**	147 (59.75)	49 (33.34)	117.08±8.41	59.0	0.76	1(Ref)		0.71
**ER negative**	99 (40.24)	30 (30.30)	82.06±4.91	57.0		1.08	0.68–1.72	
**PgR Status**								
**PgR positive**	141 (57.31)	50 (35.47)	113.06±8.43	43.0	0.47	1(Ref)		0.47
**PgR negative**	105 (42.68)	29 (27.62)	94.74±5.42	59.0		0.84	0.533–1.33	
**HER2/neu receptor status**								
**HER2/neu+ve**	130 (52.84)	32 (24.62)	128.03±9.87	58.0	0.002*	1(Ref)		0.001*
**HER2/neu -ve**	91 (36.99)	40 (43.96)	73.06±5.03	57.0		2.16	1.34–3.46	
**NA**	25 (10.16)	7 (28.0)	100.75±8.85	56.0				
**Triple Receptor status(ER, PgR and HER2/neu)**								
**Other combinations**	175 (71.13)	55 (31.43)	117.00±8.45	59.0	0.47	1(Ref)		0.001*
**Negative**	46 (18.69)	17 (36.96)	100.75±8.85	57.0		1.22	1.33–3.23	
**NA**	25 (10.16)	7 (28.0)	114.55±7.24	56.0				
**Metastasis**								
**No metastasis**	162 (65.85)	38 (23.46)	136.88±8.15	56.0	0.001*	1(Ref)		0.003*
**Metastasis yes**	84 (34.14)	41 (48.90)	76.28±7.18	57.0		2.07	1.26–3.05	

5 years OS in months; Log Rank p- values by mantle COX regression. p value <0.05 Significant

^a^HR: Hazards ratio,

^b^95% Class interval,

*p value <0.05 Significant

### In silico analysis

**Fig 5:**
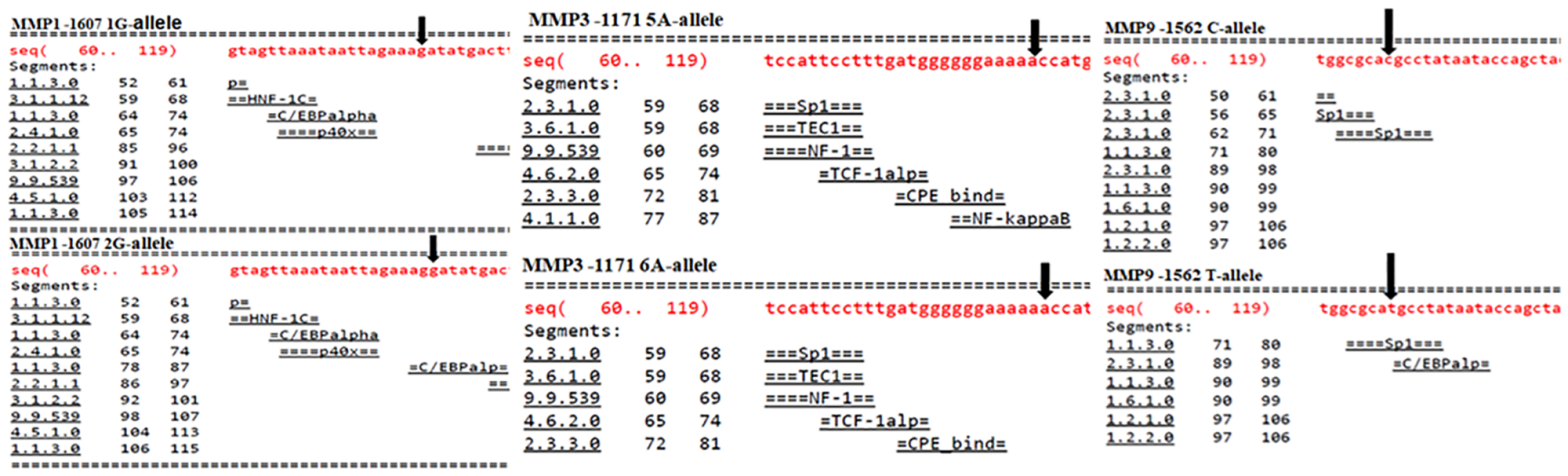
Effect of the MMP-1, -3 & -9 polymorphisms on transcription factor binding sites.

The prediction of transcription factors binding sites (TFBSs) for MMP-1–1607 1G>2G polymorphism has shown that 1G allele has no TFBS whereas 2G allele has a binding site for C/EBP alpha (CCAAT/enhancer-binding protein alpha) site as depicted in Fig 5. With respect to MMP3–1171 5A>6A polymorphism, our analysis revealed that 5A-allele has Nf-kappaB TFBS while 6A allele results in loss of Nf-kappaB binding site as depicted in Fig 5. Similarly, for MMP9–1562 C>T polymorphism, C-allele has a Sp1 TFBS, whereas T-allele results in the loss of Sp1 TFBS as shown in the Fig 5.

## Discussion

Carcinogenesis is a growing health problem worldwide that is characterized at cellular level by self-sufficiency in growth signals, insensitivity to growth-inhibitory signals, evasion of programmed cell death, limitless replicative potential, angiogenesis, tissue invasion, and metastasis. [24]. MMPs are proteolytic enzymes that degrade extracellular matrix and basement membrane. Most of the studied SNPs have been reported to have functional [25–27] a role in breast cancer progression. MMP-1 (interstitial collagenase) and MMP-3 (stromelysin) are multifunctional enzymes and structurally related genes localized to chromosome 11q, involved in physiological and pathological tissue remodeling [28]. MMP3 is known to lyse basal membrane collagen and induce the synthesis of other MMPs such as MMP1 and MMP9 (Gelatinase B) [29]. MMP9 a key player in angiogenesis, is present on chromosome 20q. The present study mainly focused on the analysis of synergistic effects of three functional SNPs of MMP1 (rs1799750) -1607 1G/2G, MMP3 (rs35068180) -1171 5A/6A and MMP9 (rs3918242) -1562 C/T genes in relation to breast cancer development and progression.

The promoter genetic variation of MMP-1 gene arises from insertion or deletion of a guanine nucleotide (G) at position -1607 relative to the transcriptional start site; consequently, one allele (insertion) has two Gs (2G), whereas the other allele (deletion) has only one G at this position (1G). The insertion creates the core sequence (5′-GGA-3′) of a binding site for the Ets transcription factors, and it was demonstrated in vitro that the 2G allele had a higher transcriptional activity [30]. In the present study as summarized in Table 4, the frequency of 2G-allele was found to be predominant in breast cancer group compared to controls, with 2 folds increased risk for BC our results are in absolute conformity with published earlier data in colorectal carcinoma and renal cell carcinoma [31]. Further 2G-allele was also found to be associated with invasiveness of lung cancer and endometrial cancer [32].

In contrast the insertion of an adenosine in the -1171 position in the MMP-3 gene promoter sequence halves its transcriptional activity [33]. It is known that the higher transcriptional activity associated with the 5A allele may enhance tumor invasiveness [34]. Our study revealed that an individual with 5A/6A genotype has an increased risk for the development of breast cancer (*p* = 0.01).

Our findings are in accordance with earlier studies carried out on non-small cell lung carcinoma in north china population [35] and early stage oral sub-mucous fibrosis, head and neck carcinoma [36]. Further, our findings are in agreement with meta-analysis report on matrix metalloproteinase 1& 3 and cancer risk [37].

MMP9 promoter region with a C to T substitution at position -1562 in MMP-9 promoter region may result in a loss of binding of a nuclear protein with increase in transcriptional activity [38]. With respect to MMP9–1562 C/T promoter polymorphism, our study revealed that the frequency of T-allele was found to be predominant in breast cancer group compared to controls, with a 1.44 folds increased risk for BC. These reports were in accordance with that of multiple cancers like lung [39] and colorectal cancer [40]. Further, Przybylowska et al (2006) has shown that the T-allele of MMP9–1562 C/T was associated with the tumor expression and influences the malignant potential of breast carcinoma susceptibility [38].

Overexpression of MMP1, MMP3 and MMP9 genes have been found to be positively associated with the clinicopathological characteristics of several malignancies [41–44]. In the present study SNPs of MMP1, MMP3 and MMP9 genes were correlated with clinicopathological features for their association in breast cancer progression and susceptibility. Our results revealed a significant association of 2G/2G genotype of MMP1-1607 with steroid hormonal receptor status and metastasis, suggesting the importance of 2G/2G genotype in the progression of breast cancer. The MMP3-1171 5A/6A polymorphism, 5A/6A genotype was significantly associated with positive lymph node involvement.

The LD analysis was carried for both controls and BC patients independently to determine the risk conferring genetic markers. The SNP loci combination was in perfect LD (D' = 1), demonstrating their strong association. Haplotype analysis of the MMP gene cluster on chromosome 11q has shown a significant association with 1G-5A variant alleles of MMP1 and MMP3 gene polymorphisms.

Further, the MDR analysis was carried out to study gene–gene interactions, the result of the present study suggests that the 2G-allele of MMP1-1607 1G/2G, and T-allele of MMP9-1562 C/T may be associated with altered enzyme activity, favours tumour-related mechanisms, and promotes tumor development and progression.

The interaction dendrogram also further confirmed that MMP-1607 1G/2G and MMP3–1171 5A/6A SNPs have strong correlation whereas, MMP9-1562 C/T polymorphism has an additive effect on the risk of breast cancer development.

Furthermore, the SNPs in promoter region of MMP-1,-3 and-9 genes might be involved in gain or loss of potential transcription factor binding sites (TFBSs), therefore we analysed the TFBSs for the SNPs under study using AliBaba2.1 online tool. Our results revealed that for MMP1–1607 1G>2G polymorphism, the 2G allele was associated with transcription factor binding sites for C/EBPalpha (also known as CCAAT/enhancer-binding protein alpha), leading to enhanced activity of the gene. Similarly, regarding MMP3–1171 5A>6A polymorphism, 5A allele was associated with Nf-KappaB site, while 6A allele was associated with lack of Nf-kappaB site, leading to reduced transcription activity. Likewise, pertaining to MMP9–1562 C>T polymorphism, C allele was associated with Sp1 nuclear protein while, T allele was associated with lack of Sp1 site, leading to increased activity of the gene.

In addition, the associations of MMP1, 3 and 9 SNPs with 5- years survival rate were assessed using Kaplan-Meier analysis. Our data showed a decreased survival rate for risk genotypes of all selected SNPs of MMP-1, -3 & -9 genes but statistically insignificant at p<0.05. However, a significant association of the clinicopathological characteristics such as late stage at diagnosis, HER2/neu receptor negative status and presence of metastasis with 5- years survival rate was observed (p<0.05), suggesting the importance of these clinicopathological features in the progression of breast cancer.

Overall, our results revealed that the polymorphisms in the promoter region of MMP1, MMP3 and MMP9 when correlated with clinicopathological characteristics and survival rate have shown significant effects on the risk and progression of breast cancer, substantiated by *in-silico* analysis. These functional polymorphisms in MMPs could lead to altered gene expression, subsequently creating imbalance in the vital MMP system that results in excessive ECM degradation and deregulated ECM dynamics in cancer development.

In conclusion, our results suggest that MMP1–1607 1G/2G, MMP3–1171 5A/6A and MMP9–1562 C/T gene polymorphisms have strong correlation with breast cancer. The 2G-6A haplotype (minor alleles of MMP-1 and MMP-3 respectively) has shown an increased susceptibility to BC, may display potential application as biological marker for identification of individuals at risk. The interactions of MMPs with breast cancer related environmental and clinical risk factors such as lymph node involvement have a strong correlation and influence the survival rate, suggesting their potential role in the breast carcinogenesis.

To the best of our knowledge this is the first study reporting on the synergistic effects of SNPs in MMP1, MMP3, and MMP9 genes in correlation with epidemiological and clinical variables also with LD, MDR, survival rate and *In-silico* analysis. However, our study has several limitations. Firstly, a small study that was analysed in South Indian population, because we restricted the study subjects to individuals of South Indian ethnicity; it is uncertain whether these results can be generalized to other populations. Second, there were few patients with missing hormonal receptor status, which may bias the results indicating an association with advanced disease status. Third, our LD and MDR analysis included only 600 samples and this may have limited the power of the pooled results. Therefore, collaborative studies on different populations are necessary to corroborate our findings.

## Supporting information

S1 File Supporting information database file (6 excel sheets titled- Control data, BC data, MDR data, LD controls, LD BC cases and SNP info). (XLS)

S2 File Supporting information database file (1 excel sheet titled- Kaplan-Meier survival data). (XLSX)

## Abbreviations

AS: allele specific
BC: breast cancer
ECM: extracellular matrix
LD: linkage disequilibrium
MDR: multifactor dimensionality reduction
MMP: matrix metalloproteinase
PCR-RFLP: polymerase chain reaction-restriction fragment length polymorphism
SNP: single nucleotide polymorphism
TIMP: tissue inhibitors of MMP
